# Prehabilitation programs – a systematic review of the economic evidence

**DOI:** 10.3389/fmed.2023.1281843

**Published:** 2023-12-01

**Authors:** Yuhe Ke, Roderica Rui Ge Ng, Shalini Elangovan, Yun Hao Leong, Zhao Han Goh, Nicholas Graves, Nicholas B. Shannon, Hairil Rizal Abdullah

**Affiliations:** ^1^Department of Anaesthesiology and Perioperative Medicine, Singapore General Hospital, Singapore, Singapore; ^2^Health Services & Systems Research, Duke-NUS Medical School, Singapore, Singapore; ^3^Duke-NUS Medical School, Singapore, Singapore; ^4^Department of General Surgery, Singapore General Hospital, Singapore, Singapore

**Keywords:** perioperative optimization, prehabilitation, cost and cost analysis, preoperative care, economic evaluation

## Abstract

**Introduction:**

Prehabilitation, which involves improving a patient’s physical and psychological condition before surgery, has shown potential benefits but has yet to be extensively studied from an economic perspective. To address this gap, a systematic review was conducted to summarize existing economic evaluations of prehabilitation interventions.

**Methods:**

The PRISMA Protocols 2015 checklist was followed. Over 16,000 manuscripts were reviewed, and 99 reports on preoperative interventions and screening tests were identified, of which 12 studies were included in this analysis. The costs are expressed in Pounds (GBP, £) and adjusted for inflation to December 2022.

**Results:**

The studies were conducted in Western countries, focusing on specific surgical subspecialties. While the interventions and study designs varied, most studies demonstrated cost savings in the intervention group compared to the control group. Additionally, all cost-effectiveness analysis studies favored the intervention group. However, the review also identified several limitations. Many studies had a moderate or high risk of bias, and critical information such as time horizons and discount rates were often missing. Important components like heterogeneity, distributional effects, and uncertainty were frequently lacking as well. The misclassification of economic evaluation types highlighted a lack of knowledge among physicians in prehabilitation research.

**Conclusion:**

This review reveals a lack of robust evidence regarding the economics of prehabilitation programs for surgical patients. This suggests a need for further research with rigorous methods and accurate definitions.

## Introduction

Prehabilitation was first proposed as part of the Enhanced Recovery Programme (ERAS) in 1997 (1). It involves a range of interventions, including physical activity, nutrition (2), and psychological and educational interventions; all designed to improve a patient’s physical and psychological condition before surgery. It addresses the issue of malnutrition and sarcopenia, which is endemic in the surgical population (3). Prehabilitation, especially exercise interventions, offers a range of significant benefits that can be broadly classified into three main areas. Firstly, it empowers individuals by enhancing their sense of control and purpose, ultimately leading to an improved quality of life. Secondly, it has proven to enhance physiological and psychological resilience, thereby improving the overall quality of recovery. Lastly, prehabilitation promotes positive long-term behavioral changes, which are likely to result in sustained health benefits (4). As such, prehabilitation has been found to improve perioperative functional capacity for patients undergoing major abdominal surgery (5), reducing the risk of postoperative complications and improving outcomes (6–9).

Despite some promising findings, previous systematic reviews of prehabilitation programs revealed heterogeneity in their composition, mode of administration, and outcome measures of functional capacity used to evaluate impact (10–12). Additionally, randomized controlled trials on prehabilitation have yielded controversial results, where some studies demonstrate improved patient outcomes (13, 14), while others report no significant differences in outcomes when compared to control or rehabilitation groups (15, 16). These variabilities and inconsistencies in research outcomes may be a significant factor contributing to the challenge of establishing standardized prehabilitation programs worldwide despite increasing research since the inception of the ERAS movement (17, 18).

One of the main challenges is the large cost associated with implementation, which likely resulted in intermittent adoption, especially in low- and middle-income countries (19). According to the United Kingdom National Health Service (NHS) 2020/21 National Cost Collection Data Publication, outpatient follow-up physiotherapy, dietitian, and education services cost £116, £110, and £210 per visit, respectively, in the NHS (20). Outpatient multidisciplinary care, including a review by a geriatrician, averages £386 per visit (20). Furthermore, there have been concerns about the efficacy of preoperative care for all preoperative patients (21).

While prehabilitation might appear relatively costly, it is important to consider the potential effect on downstream costs and health outcomes when deciding whether to invest in these interventions. The savings from prehabilitation, such as reduced length of hospital stay and complication rates (6–9), may ultimately outweigh the implementation cost. With an estimated 300 million surgeries performed worldwide annually (19), evaluating the broader economic impact of prehabilitation programs is important.

Economic evaluations can reveal whether the adoption of an intervention is likely to improve the efficiency of spending on health services. Frequently used methods are cost-only analysis (CA), cost-effectiveness analysis (CEA), cost-utility analysis (CUA), and cost–benefit analysis (CBA). CA compares only the costs of alternative interventions and is a partial evaluation. CEA estimates the monetary value of costs and assesses the intervention’s impact on a specific measure of effect, in natural units of an outcome. CUA is a special form of CEA and employs the quality-adjusted life years (QALYs) to represent the health benefits of interventions. CBA assesses the willingness to pay, in monetary terms, of stakeholders for the perceived benefits of the intervention; both costs and benefits are in monetary terms. While CA is not always informative for decision-making as health effects are ignored, CEA, CUA, and CBA are more likely to provide information that is useful for decision-making. Table 1 provides an overview of the four types of economic analyses (22–24).

**Table 1:** Types of economic evaluations in healthcare.

Types	Intervention(s)/policies	Measurement in healthcare effects	Advantages and disadvantages
Cost analysis (CA)	Evaluates two or more policies by their impact on costs only	N/A	- Simpler to conduct- Fails to reveal the impact on health outcomes
Cost-effectiveness analysis (CEA)	Comparison of two or more policies that produce a common unit of effect	Health effects measured in natural units of outcome	- Shows how to provide natural units of health outcome at minimum cost- Unable to compare across different programs that report different effects
Cost-Utility analysis (CUA)	Comparison of two or more policies to reveal morbidity and/or mortality benefits.	Preference-based health outcomes such as QALY.	- Shows how to provide incremental QALYs at minimum cost- Enables comparison across different programs and patient groups
Cost–benefit analysis (CBA)	Evaluates two or more policies where the individual preferences of stakeholders are included	Monetary values derived from contingent valuation studies	- Enables direct measurement of preferences of stakeholders- Difficult to elicit valid responses- Income effects can bias results

QALY, Quality-adjusted life years.

The current understanding of the economic impact of preoperative interventions is limited and lacks comprehensive research (25), and there is a significant knowledge gap in the economic evidence of prehabilitation programs. The aim of this systematic review is to summarize the current economic evaluation research for prehabilitation programs. We intend to provide insights for decision-makers about the potential economic outcomes of implementing these programs into clinical practice.

## Methods

The Preferred Reporting Items for Systematic Reviews and Meta-Analyses (PRISMA) Protocols 2015 checklist (26) was adhered to in this systematic review.

### Search terms

Papers that describe economic evaluations of interventions and screenings done in the preoperative setting were selected for data extraction. There were two main steps to the search strategy. The first is to identify the publications in any preoperative interventions which address economic evaluations. The second step was to extract studies related to prehabilitation.

#### Identification of preoperative interventions

The search strategy was conducted in three main components: official terms for preoperative care OR a combination of terms that may indicate different variations of preoperative anesthesia clinic (e.g., pre-anesthesia, anesthesia, preoperative evaluation, preadmission, surgical procedures, elective) AND costs and cost analysis (e.g., Health Services Research, Health Resources, Delivery of Health Care) which would include all forms of economic analysis. The searches were conducted in MeSH terms and titles and all fields for the PubMed extraction. The detailed search term for each category can be found in Appendix Table A. For the other databases, variations of terms will be used to extract from the title and keywords.

A total of 99 papers on economic analysis of preoperative interventions were identified via this search strategy. The paper titles and abstracts were reviewed by the authors and the further subtopic of prehabilitation was identified as one of the main themes and is reported in this paper.

#### Identification of prehabilitation papers

Studies, where interventions included any form of preoperative prehabilitation (nutrition, physiotherapy, medical optimization and education), were selected after a review of abstracts. Studies were excluded if they did not separate preoperative and postoperative components, such as a blanket ERAS protocol which also involved intraoperative and postoperative interventions. This is not to repeat previous works on ERAS (27).

### Databases searched

A selection of economic evaluation analyses published between 1996 (28) and 1st December 2022 was obtained from the following database: PubMed, Embase (Ovid), Web of Science, Cost-Effectiveness Analysis (CEA) Registry, the Cochrane Database of Systematic Reviews, China National Knowledge Infrastructure (CNKI). The CEA registry is an open-access international repository of systematically reviewed healthcare cost-effectiveness analyses. This database can be accessed and searched through www.cearegistry.org and sponsors of the database can download it as a spreadsheet. The CNKI registry is a Chinese database registry that provides a complete collection of China journals.

### Inclusion and exclusion criteria

Papers are included if the intervention in the study is (1) conducted in the preoperative setting, (2) only contains preoperative intervention and does not contain any post-operative component, and (3) looks at either nutrition, physiotherapy, medical optimization, and education of the patient. All other interventions that do not fulfil these criteria were excluded. We also excluded analyses that were abstracts, case reports, systematic reviews, meta-analyses, comments, letters to the editor, and expert opinions, as well as interventions that cannot be performed in the preoperative outpatient clinic setting. The population where cohorts included patients younger than 18 years old and emergency surgery were excluded. Studies that were published in both English and Chinese were included.

Costs data were converted to Great Britain Pounds (GBP) based on the year that the cost was reported within the study using the historical exchange rate found at https://www.exchangerates.org.uk/ (Appendix Table B). The cost was then adjusted for inflation to the December 2022 GBP currency using the national inflation calculator for Great Britain Pounds (GBP).^1^

The RoB 2 tool (Cochrane, Denmark) was used to assess the risk of bias (29) in randomized controlled trials. The CHEERS 2022 checklist (30) was used to assess the conduct of economic evaluations in the papers.

## Results

### Overview

**Figure 1:**
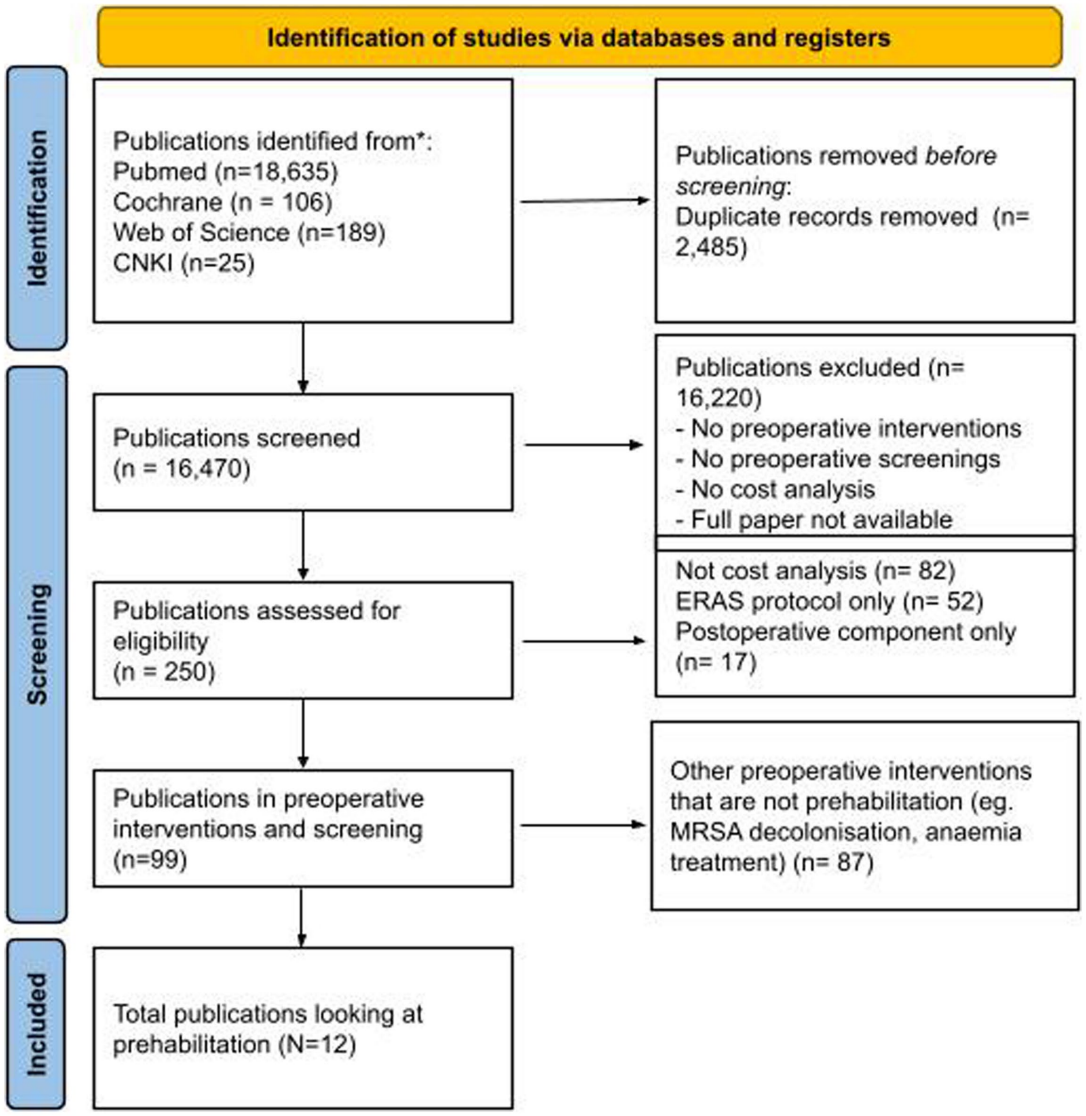
PRISMA flow diagram. A total of 12 studies were included in the final analysis.

We identified a total of 18,955 citations published between 1st Jan 1996 and 1st Nov 2022. We identified a total of 12 studies that met our inclusion criteria and were suitable for quantitative analysis (Figure 1) (31–42). A pooled total of 2,448 patients was recruited in these studies.

These studies were conducted in the United Kingdom (*n* = 4), European countries (*n* = 4), the United States (*n* = 2), Canada (*n* = 1), and Australia (*n* = 1). Only 5 of the 12 studies (41.7%) reported costs as a primary outcome. The populations included in these studies included total knee replacements (*n* = 2), major abdominal surgeries (*n* = 7), cardiothoracic operations (*n* = 2) and major vascular operations (*n* = 1), see Table 2.

**Table 2:** Characteristics of studies stratified by type of analysis performed.

Source	Study design	Total recruited	Population	Intervention	Lower cost	Cost-effective
Cost analysis						
Beaupre et al. (31)	RCT*	131	Knee arthroplasty 40–75 years old	Physiotherapy, Education	Control	
Mcgregor et al. (32)	RCT*	35	Knee arthroplasty	Education	Intervention	
Barberan-Garcia et al. (33)	RCT*	125	Major abdominal surgery >70 years old ASA 3 or 4 DASI<46	Physiotherapy, Education	Intervention	
Smedley et al. 2004 (34)	RCT*	152	Major abdominal surgery	Nutrition	Intervention	
Robinson et al. (37)	Retrospective	462	Thoracic neoplasm resection	Nutrition	Intervention	
Braga et al. (40)	RCT	92	Major abdominal surgery	Nutrition	Intervention^	
Ploussard et al. (41)	RCT	507	Robotic Radical Prostatectomy	Physiotherapy, Education, Nutrition	Intervention	
Cost-effectiveness analysis/Cost-utility analysis						
Boden et al. (35)	RCT	441	Major abdominal surgery	Education	Intervention^	Intervention
Rolving et al. (43)	RCT*	90	Lumbar spine surgery (max 3 levels) 18–64 years old	Education	Intervention^	Intervention
Partridge et al. (38)	RCT*	209	Major vascular surgery >65 years old	Medical optimization	Intervention^	Intervention
Furze et al. (39)	RCT*	204	Coronary artery bypass graft	Education	Control	Intervention
Leeds et al. (42)	Decision tree model	10,000 simulated	Colon cancer surgery	Medical optimisation	Intervention^	Intervention

Lower cost represents the group where the cost analysis was lower; cost-effective represents the group that the cost-effectiveness analysis favors. *Studies that received national funding. ^Studies where cost analysis was the primary outcome. RCT, Randomized controlled trial; N, Nutrition; P, Physiotherapy; E, Education; M, Medical Optimization.

All the studies reported cost analysis outcomes, but only 5 of the studies reported CEA/CUAs outcomes (35, 36, 38, 39, 42). Ten of the studies (83.3%) reported lower costs in the intervention group. All the CEA/CUAs studies showed that adopting the intervention was likely to be a cost-effective decision.

### Details of interventions

The studies were categorized into four subcategories: Nutrition, Physiotherapy, Education, and Medical Optimization. There were variations in how the interventions were conducted among the studies.

For preoperative nutrition, there were 4 studies that examined this aspect (34, 37, 40, 41). Two of the studies gave Oral Impact Powder, while one study gave Fortisip, and one study did not specify the brand of immunonutrition given. The duration of preoperative nutrition also ranged from 5 to 7 days. Smiley et al. (30) focused on preoperative nutrition and conducted a four-arm study, where patients received either pre- and post-operative nutrition (SS), preoperative nutrition only (SC), postoperative nutrition only (CS), or no nutritional supplementation (CC). In this case, two comparison arms were made between the SS and CS groups and the CC and SC group.

As for physiotherapy, there were 3 studies offering distinct approaches to prepare individuals for surgery (31, 33, 41). Beaupre et al. and Barberan-Garcia et al. shared a common goal of enhancing broader physical capacities, including strength and aerobic capacity. However, the modalities of exercise varied significantly between these two studies (31, 33). Beaupre et al. employed simple strength and progressive resistance training sessions, carried out 3 to 4 times per week over a duration of 6 weeks (31). In contrast, Barberan-Garcia et al. implemented a high-intensity enhanced exercise training program for a condensed 4-week period (33). Conversely, Ploussard et al. took a distinctive approach by focusing specifically on pelvic floor exercises (41). Unfortunately, the timing of the commencement of these exercises was not specified. Nevertheless, this study stands out for its specificity, recommending pelvic floor exercises to be performed 2 to 3 times daily (41).

There were 7 studies which intervened with education (31–33, 35, 36, 39, 41). All studies incorporate some form of educational material, either through booklets (32, 35, 36, 41) or direct instructions (31, 33, 39). Collectively, the education intervention was broad ranging from providing information about the surgery and expected recovery (32) to postoperative exercises (25, 31, 32) to even relaxation or pain coping strategies (36, 39).

Lastly, only two studies specifically explored the role of medical optimization in the context of prehabilitation (39, 42). Partridge et al. undertook a comparative analysis between assessments conducted by a registrar-level geriatrician in a one-stop clinic, which includes the formulation of an optimization plan by a multidisciplinary team, and assessments performed by preoperative clinic nurses following a protocolized appraisal of anesthetic and medical issues (39). In contrast, Leeds et al. focused on targeted outpatient care, where patients receive specialized attention from subspecialists such as pulmonologists or endocrinologists and compared it to cases where no targeted outpatient care is provided (42).

Details of interventions done in each study can be found in Appendix Table C.

### Cost analysis

In all studies, the intervention group demonstrated cost savings when compared to the control group, with the exception of Beaupre et al. which reported a cost increase of £3 per patient for the intervention group (31). Details of costs included for the intervention and control arms are reported in Appendix Table D.

The study with the largest cost saving was Leeds et al. with £2,673 saved per patient during the hospital stay (42). In comparison, Partridge et al. showed medical optimization with registrar-level geriatrician review had a saving of £1,268 (34). Robinson et al. (37) and Braga et al. (40) had the next highest savings after Leeds et al. (42). Both studies looked at preoperative nutritional supplementations which resulted in savings of £2,123 and £1,412, respectively. The cost ranges between each sub-category are presented in Table 3.

**Table 3:** Total cost savings categorized by prehabilitation sub-category of nutrition, physiotherapy, education, and medical optimization.

Nutrition (34, 37, 40, 41)	Physiotherapy (31, 33, 41)	Education (31–33, 35, 36, 39, 41)	Medical optimization (38, 42)
£58 – £2,123 (4 studies)	–	£91 – £1,329 (4 studies)	£1,218 – £2,673 (2 studies)
–	£-3 – £736 (2 studies)		–
£406 (1 study)			–

The costs are expressed in Pounds (GBP, £) and adjusted for inflation to December 2022.

Three studies (35, 36, 39) analyzed preoperative education interventions using CEA, which showed that the probability of the intervention being cost-effective is greater than 70% at the willingness of pay threshold set in the paper and adjusted for inflation and currency(£). Of note, Rolving et al. did not present the ICER ratio, but instead, plotted the threshold for willingness to pay and the probability of the intervention being cost-effective for both QALY gained and Oswestry Disability Index (ODI) gain by 15 points (43). Table 4 provides a detailed breakdown of the CEA analysis performed in these studies.

**Table 4:** Cost-effectiveness analysis of prehabilitation interventions.

Source	Cost difference (Mean)	Changes in QALY (Mean)	ICER	Favors
Intervention: Education				
Boden et al. (35)	-£1,329	0.020	Dominant (costs lower by $16,274 per QALY gained)	Intervention
Furze et al. (39)	-£3.92	0.006	Dominant (costs lower by $476 per QALY gained)	Intervention
Rolving et al. (43)	Not calculated (Appendix Figure A)	Intervention		
Intervention: Medical optimisation				
Partridge et al. (38)	-£1,218	0.58	Dominant (costs lower by $2,099 per QALY gained)	Intervention
Leeds et al. (42)	-£2,673	0.03	Dominant (costs lower by $89,069 per QALY gained)	Intervention

Cost difference refers to the total cost per patient of the treatment group – control group. Dominant indicates that the intervention group dominated the control group by cost and QALY outcomes. The costs are expressed in Pounds (GBP, £) and adjusted for inflation to December 2022. ICER, Incremental cost-effectiveness ratio; QALY, Quality-adjusted life years.

### Economic evaluations checklist and risk of bias

The CHEERS 2022 checklist was used to evaluate the quality of the reporting among the studies (44). It consisted of 28 items accompanied by descriptions and was updated in 2022. The method section of the checklist was evaluated for the 12 studies included (Appendix Figure A).

We found that more than half of the studies did not report critical information such as time horizons (6/12) and discount rates (8/12) in their methods. Additionally, 5 studies did not report currency, price date, and conversions. While traditional components of study methods such as study population, settings and locations, comparators and outcome measurements were present in all studies, certain components like the characterization of heterogeneity, distributional effects, and uncertainty were not present in 75% of the studies. It is worth noting that none of the studies reported the effect of engagement with patients and others affected by the study, which is an important consideration in health economic evaluations.

**Figure 2:**
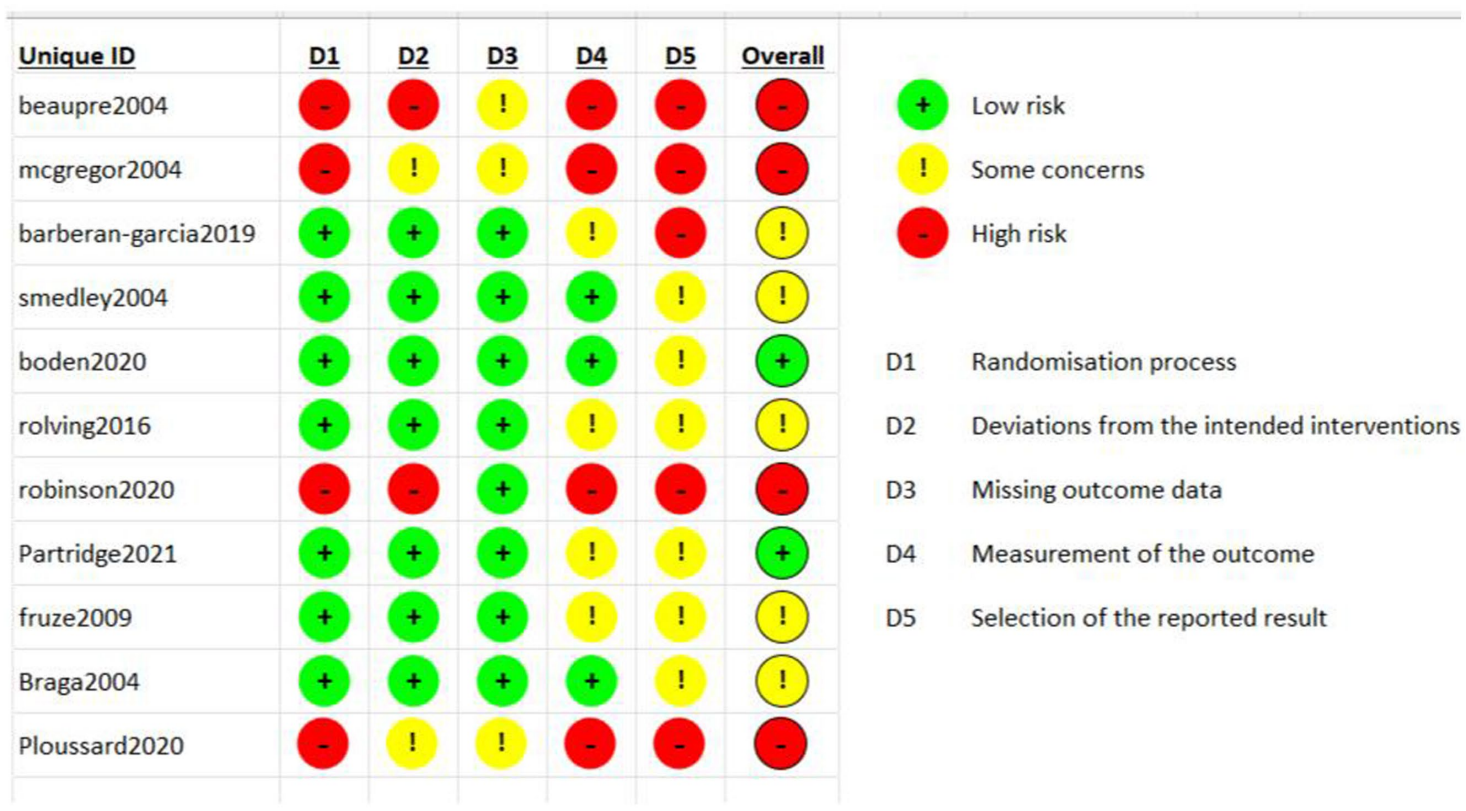
Risk of bias assessment based on version 2 of the Cochrane risk-of-bias tool for randomized trials (RoB 2).

Figure 2 shows the results of the risk of bias assessment using the RoB tool (29). Four of the studies report a high risk of bias and 5 studies reported a moderate risk of bias due to multiple outcome measurements and selected reporting of results. There were also questions about the randomization processes with some studies, especially those published in earlier years, failing to describe how the patients were allocated into different groups. Leeds et al. was not included in the assessment (42).

## Discussion

The aim of this study was to conduct a systematic review of the economic evaluations of prehabilitation interventions for surgical patients. Our findings show a limited number of economic evaluations meeting our inclusion criteria. Half of the studies were conducted more than a decade ago.

### Overall

Since the first RCT on prehabilitation in 2000 (45) in the CABG population, there have been various studies examining the benefits of prehabilitation, with the majority of them investigating the clinical outcomes (46–48). The clinical findings are largely positive (49). However, there are also opinions that the evidence for definitive clinical effectiveness is still limited (50). A Cochrane review of 4 RCTs on prehabilitation exercise therapy before elective abdominal aortic aneurysm repair also shows that there is poor evidence to suggest that prehabilitation exercise therapy reduces 30-day mortality, pulmonary complications, need for re-intervention or postoperative bleeding (48). With the exorbitant cost of prehabilitation (ranging from £109.91 for an outpatient dietician and £385.93 for geriatrics assessments (20)), it is imperative that high-quality economical evaluations for prehabilitation interventions are done to justify the cost-effectiveness for wider implementation.

This systematic review showed that all the studies on the economic evaluation of prehabilitation were conducted in western countries and various subspecialties of surgical patients were studied. The interventions varied across studies even among the same sub-category, which contributed to the heterogeneity of the findings. Most of the economic analyses in this study were conducted as secondary analyses to RCTs. While this approach can be useful in providing additional economic information to support the clinical trial findings, the small sample sizes of the economic analyses may limit the generalizability and have various other limitations (details below).

### Preoperative nutrition

Four studies investigated the impact of preoperative nutrition (34, 37, 40, 41). These studies, encompassing patient cohorts undergoing surgery for thoracic neoplasms, major abdominal surgeries, and robotic prostate surgery, consistently revealed cost-savings though to varying extents. However, a recent systematic review focusing on oral nutrition in frail elderly individuals who were malnourished or at risk of malnutrition found limited evidence supporting its benefits (51). While this review did not specifically target the preoperative cohort, it serves as a timely reminder that interventions may not yield the same cost-effective results across different types of surgery and patient populations. Despite the variability in evidence, it is notable that the improvement in patient outcomes is the most pronounced in head and neck oncological surgeries and gastrointestinal (esophageal, gastric, colonic) surgeries, hence early and appropriate nutritional support should be prioritized for these at-risk patients (52).

### Preoperative physiotherapy

The CA studies generally favored the intervention group, with the exception of Beaupre et al. (31), where the overall costs were higher for patients who received preoperative physiotherapy and education. It is worth noting that this study specifically focused on knee replacement arthroplasty in a younger population, which may explain the lack of cost–benefit compared to other studies where more major operations in a sicker patient population were examined. Other systematic reviews on prehabilitation physiotherapy have also questioned the effectiveness of these exercises in reducing 30-day mortality and pulmonary complications (48). This highlights that prehabilitation may not be cost-effective in all surgical populations. However, the most consistent evidence suggests that preoperative exercise has a modest yet positive effect on postoperative pain and functional outcomes at 6 months for individuals undergoing joint replacement surgery (53).

### Preoperative education

Preoperative education was conducted and studied most frequently in this literature review. The education interventions included (1) preoperative optimization such as physiotherapy (33, 34, 39, 41), (2) intraoperative expectations (32), and (3) expectations of postoperative recovery (31, 32, 35, 39). Rolving et al. even discussed postoperative pain coping strategies during preoperative education (36). One of the significant benefits of preoperative education is that it empowers patients to become more confident in their ability to carry out perioperative tasks and be motivated to improve their preoperative status (54). The CEAs performed show that preoperative education is likely to be cost-effective.

### Preoperative optimization

Preoperative optimization from interdisciplinary healthcare consultations is potentially cost-effective in targeted patient groups (38, 42). While the cost of outpatient specialist visits can be significant, referring patients to various specialists can result in goal-driven interventions that ultimately improve postoperative outcomes (55), highlighting the importance of multidisciplinary care in the perioperative setting. By implementing a collaborative approach that includes medical specialists, nurses, and other healthcare professionals, preoperative optimization can be tailored to the individual needs of the patient, resulting in better outcomes and lower costs in the long run. Briggs et al. also demonstrated that cost analysis can also be calculated directly with economical evaluation modeling (56).

### Lack of knowledge of the terminology for economic evaluation

This review highlights a lack of high-quality evidence available on prehabilitation interventions and insufficient knowledge among physicians regarding the terminology for economic evaluations. One example of this is the misleading title of the Braga et al. study (40), which claimed to be a “cost–benefit analysis” but only reported a *CA*.

The evaluation of the CHEERS checklist further revealed deficiencies in economic studies. Specifically, the majority of studies did not characterize how the study would vary with different subgroups, how impacts are distributed across different individuals, or characterize sources of uncertainty without the analysis. Additionally, the studies failed to describe any approaches to engage patients and stakeholders in the design of the study.

### Limitations

Nearly all of the economic analyses were conducted within the context of RCTs. While RCTs offer high internal validity by adhering to strict protocols, they may not always reflect real-world scenarios, limiting their external validity. As a result, the hierarchy of evidence for economic evaluations may differ from that of other types of research. While RCTs provide important insights into the effectiveness and cost-effectiveness of interventions, it is crucial to consider the limitations and potential biases inherent in their design when applying their findings to broader populations or clinical settings. There is also a limited number of studies with heterogeneous interventions and the overall confidence of evidence needs to be interpreted with caution.

### Future works

While this systematic review contributes valuable insights into the economic evaluations of prehabilitation interventions for surgical patients, several avenues for future research and improvement in methodology emerge.

Firstly, considering the limited number of economic evaluations meeting inclusion criteria and the predominantly Western focus of the studies, there is a need for more studies from diverse geographical regions to enhance the generalizability of findings. Secondly, the heterogeneity in interventions within the same sub-categories calls for standardized approaches and protocols in prehabilitation studies. Developing consensus on intervention components and delivery methods would facilitate more accurate comparisons and generalizable conclusions. Additionally, given the evolving landscape of prehabilitation research, there is a need for more recent economic evaluations, as nearly half of the studies included in this review were conducted more than a decade ago (31, 32, 34, 39, 40). Furthermore, focusing on a more diverse range of surgical populations and exploring the impact of prehabilitation on different types of surgeries could provide nuanced insights. Finally, future research should strive to bridge the knowledge gap among physicians regarding the terminology for economic evaluations, ensuring accurate and transparent reporting to facilitate better comprehension and application of study findings.

Addressing these aspects would contribute to a more comprehensive understanding of the economic aspects of prehabilitation interventions and guide their effective implementation in diverse clinical settings.

## Conclusion

The findings of this systematic review indicate a scarcity of high-quality evidence concerning the cost-effectiveness of prehabilitation programs. However, there is some suggestion that preoperative education and medical optimization interventions may offer cost-effective benefits. Further research is warranted to gain a comprehensive understanding of the economic impact of prehabilitation programs. Additional studies are needed to elucidate the potential economic advantages of implementing prehabilitation programs and to guide decision-making in healthcare settings.

## Data availability statement

The original contributions presented in the study are included in the article/Supplementary material, further inquiries can be directed to the corresponding author.

## Author contributions

YK: Conceptualization, Data curation, Methodology, Formal analysis, Writing - original draft, Writing - review & editing. RN: Conceptualization, Methodology, Formal analysis, Writing – original draft, Writing – reviewing & editing. SE: Methodology, Writing – review & editing. ZG: Methodology, Validation, Writing – review & editing. YL: Validation, Writing – review & editing. NG: Writing – review & editing. NS: Methodology, Software, Visualization, Writing – review & editing. HA: Funding acquisition, Supervision, Writing - review & editing.

## Footnotes

^1^https://www.bankofengland.co.uk/monetary-policy/inflation/inflation-calculator
