# Supplementary material for: Prehabilitation programs – a systematic review of the economic evidence
Source: Front Med (Lausanne). 2023 Dec 1;10:1281843. doi: 10.3389/fmed.2023.1281843 (PMC10722222; doi:10.3389/fmed.2023.1281843)
Supplement: Supplementary file 1 [file Data_Sheet_1.docx]

Appendix

Table A: Search terms for systematic review stratified by databases.

| Pubmed |
| --- |
| #1 "Preoperative care"[Mesh] OR "Preoperative Period"[Mesh] OR "Perioperative Period"[Mesh] OR ”Outpatient Clinics, Hospital” [Mesh] #2 “preoperative evaluation”[All Fields] OR “pre-anaesthesia”[All Fields] OR “anesthesia”[All Fields] OR “‘preoperative assessment”[All Fields] OR “preadmission”[All Fields] OR “preoperative centre”[All Fields] OR “preoperative unit”[All Fields]  #3 ("surgical procedures, elective"[MeSH Terms] OR ("surgical"[All Fields] AND "procedures"[All Fields] AND "elective"[All Fields]) OR "elective surgical procedures"[All Fields] OR ("elective"[All Fields] AND "surgery"[All Fields]) OR "elective surgery"[All Fields])  #4 ("Costs and Cost Analysis"[Mesh] OR "Health Services Misuse"[Mesh] OR "Health Resources"[Mesh] OR "Delivery of Health Care"[Mesh] OR "Health Services Research"[Mesh])  #5 (#1 and #4)  #6 (#2 and #4)  #7 (#3 and #4)  #8 “preoperative” [Title] OR “anesthesia”[Title]  #9 “Cost utility”[Title] OR “cost effectiveness”[Title] OR “economics”[Title]  #10 (#8 and #9) |
| Embase (Elsevier) |
| #1 ‘preoperative care’/exp OR ‘preoperative care’  #2 'elective surgery'/exp OR ‘preoperative evaluation’ OR ‘preoperative assessment’  #3 ‘Costs’/exp economic aspect OR ‘Economics’ cost:.tw. OR ‘Çosts’ cost effective:.tw. [(McKinlay et al. 2006)](https://paperpile.com/c/qtvVUH/4Oev)  #4 (#1 AND #2 AND #3) |
| Web of Science (Current Contents Connect) |
| #1 TOPIC: ’preoperative care’ OR ‘preoperative period’  #2 TOPIC: ’elective surgery’ OR ‘preoperative evaluation’ OR ‘preoperative assessment’  #3 TOPIC: ’economics’ OR ‘cost effectiveness’ OR ‘cost utility’  #4 (#1 AND #3)  #5 (#2 AND #3) |
| CEA registry [(CEA registry )](https://paperpile.com/c/qtvVUH/EXr7) |
| #1 Title: ’preoperative care’ OR ‘preoperative period’ OR ‘anesthesia’  #2 Title: ’elective surgery’ OR ‘preoperative evaluation’ OR ‘preoperative assessment’ |
| Cochrane Database of Systematic Reviews |
| #1 Title abstract keywords: ’preoperative care’ OR ‘preoperative period’  #2 Title abstract keywords: ’elective surgery’ OR ‘preoperative evaluation’ OR ‘preoperative assessment’  #3 Title abstract keywords: ’economics’ OR ‘cost effectiveness’ OR ‘cost utility’  #4 (#1 AND #3)  #5 (#2 AND #3)  #6 ("Preoperative care"[Mesh] OR "Preoperative Period"[Mesh] OR "Perioperative Period"[Mesh] OR ”Outpatient Clinics, Hospital” [Mesh])  #7 ("Costs and Cost Analysis"[Mesh] OR "Health Services Misuse"[Mesh] OR "Health Resources"[Mesh] OR "Delivery of Health Care"[Mesh] OR "Health Services Research"[Mesh])  #8 (#6 AND #7) |
| China National Knowledge Infrastructure |
| #1 Subjects: 围手术期 (Perioperative) OR 手术前 (Preoperative) OR 麻醉 (Anesthesia) OR 加速康复外科 (ERAS)  #2 Subjects: 经济效果 (Economics) OR 成本效益 (Cost analysis) OR 经济效益 (Cost utility and effectiveness)  #3 (#1 AND #2) |

**Table B:** Conversion table used to convert to pounds (GBP).

| **Source** | **Year of cost reporting** | **Currency** | **Conversion to Pound (Average closing price)** |
| --- | --- | --- | --- |
| Beaupre et al | 1997 | CAD | 1 CAD = 0.4433 GBP |
| Mcgregor et al | 2003* | GBP | - |
| Barberan-garcia et al | 2017 | EUR | 1 EUR = 0.8766 GBP |
| Smedley et al | 2004* | GBP | - |
| Boden et al | 2018 | AUD | 1 AUD = 0.5953 GBP |
| Rolving et al | 2014 | ERO | 1 EUR = 0.8061 GBP |
| Robinson et al | 2018* | USD | 1 USD = 0.7501 GBP |
| Partridge et al | 2018 | GBP | - |
| Furze et al | 2004 | GBP | - |
| Braga et al | 2004* | ERO | 1 EUR = 0.6822 GBP |
| Ploussard et al | 2019* | ERO | 1 EUR = 0.8773 GBP |
| Leeds et al | 2021 | USD | 1 USD = 0.7271 GBP |

*Year of cost reporting was not made clear in the paper. As such, the last year of patient recruitment was used.

**Table C:** Details of the prehabilitation interventions and controls that were conducted in the studies. The interventions are divided into those targeted at 1) Nutrition, 2) Physiotherapy, 3) Education and 4) Medical Optimisation.

| **Source** | **Intervention Group** | | | | **Control Group** |
| --- | --- | --- | --- | --- | --- |
|  | **1) Nutrition** | **2) Physiotherapy** | **3) Education** | **4) Medical Optimisation** |  |
| Beaupre et al |  | Initial contact: 6 weeks prior to operation  Target: Improve knee mobility and strength  Duration: 3 times/week for 4 weeks  Details: Simple strength training and progressive resistance training. | Instructions on:   - Crutch walking on ground level and stairs - Bed mobility and transfers - Postoperative exercises |  | No exercise therapy  No education |
| Mcgregor et al |  |  | Advice class held 2-4 preoperative operatively  Information Booklet   - Stages of surgery and expected recovery - Postoperative exercises - Use of walking aids |  | No booklet  Standard preoperative consenting |
| Barberan-garcia et al |  | Initial contact: 4 weeks prior to operation  Target: Increase aerobic capacity and enhance physical activity  Duration: At least 4 weeks  Details: High-intensity enhance-exercise training programme | Motivational interview and promotion of physical activities at the start of training programme |  | Standard preoperative care including:   - Counselling on physical activities, nutritions, smoking and alcohol cessation - IV iron for iron deficiency anemia |
| Smedley et al | Preoperative Oral nutrition only (SC):  Nutrition: Fortisip(1·5 kcal and 0·05 g protein/mL)  Frequency: Drink in small amounts between meals  Duration: At least 7 days before operation to 24 hrs before operation.  ----  Preoperative oral nutrition and postoperative nutrition (SS) |  |  |  | No pre- and post-operative nutrition (CC).  ------  Only postoperative nutrition (CS)  Nutrition: Fortisip(1·5 kcal and 0·05 g protein/mL)  Frequency: from first day patient can tolerate free fluids/light diet  Duration: 4 weeks |
| Boden et al |  |  | Assessment by physiotherapist:   - Respiratory, social and functional assessment   Educated on deep breathing exercises and coughing  Information booklet |  | No physiotherapy visit  No information booklet  Standard postoperative early ambulation programme |
| Rolving et al |  |  | Group-based education sessions on:   - Pain behaviour and pain coping strategies   Total 6 sessions |  | No group-based education session |
| Robinson et al | Advanced Recovery Frequency (immunonutrition supplement  Impact)  Frequency: TDS for 5 days  ClearFast (complex carbohydrate)  Frequency: 7pm the day before surgery and 4.30am on day of surgery  Daily multispecies probiotics  Frequency: From clinic visit to surgery |  |  |  | No nutritional supplementations |
| Partridge et al |  |  |  | Assessment by registrar-level geriatrician:  One-stop clinic with the formulation of optimisation plan by multidisciplinary team | Assessment by preoperative clinic nurses:  Conduct protocolised  appraisal of anaesthetic and medical issues |
| Furze et al |  |  | HeartOp Programme:   - Education booklet on postoperative complications risk factors - Relaxation programme - Goal setting to reduce CVS risk and increase activity level. |  | No effort to elicit certain misconceptions.  General advice is given instead of specific goal setting. |
| Braga et al | Preoperative oral impact (immunonutrition supplement)  Frequency: 5 days before operation |  |  |  | No preoperative nutrition |
| Ploussard et al | Oral nutrition supplementations for underweight patients.  Weight loss for overweight patients.  Dietitian review. | Presurgical pelvic floor exercises 2 to 3 times a day | Information booklet:   - Walking programs - Aerobic training - Cardiorespiratory fitness |  | With just ERAS protocol |
| Leeds et al |  |  |  | Targeted outpatient care by a subspecialist (e.g.,  pulmonology, endocrinology) | No targeted outpatient care |
| Total | 4 +1 | 3 | 7 | 2 |  |

**Table D:** Cost-only analysis with differences of total cost per patient adjusted for inflation to December 2022 (GBP).

| Source | **Cost-only analysis: Total cost per patient (GBP)** | | |
| --- | --- | --- | --- |
|  | **Intervention: Nutrition** | | |
|  | **Treatment** | **Control** | **Difference**  **(Treatment - Control)** |
| Smedley,  Mean(SD) | 3,784 (3,393, 4,248) (SC) | 4,334 (3,761, 5,266) (CC) | -550 |
| Smedley,  Mean(SD) | 3,789 (3,367,4,498) (SS) | 3,847 (3,340,4,405) (CS) | -58 |
| Robinson  Mean(SD) | 11,124 (4,396) | 13,247 (6,919) | -2,123 |
| Braga  Mean | 2,114 | 3,526 | -1,412 |
|  | **Intervention: Physiotherapy + Education** | | |
| Beaupre,  Mean | 950 | 947 | 3.31 |
| Barberan-garcia,  Mean (Min-Max) | 4,044 (1,335, 22,746) | 4,780 (1,182 - 2,5718) | -736.34 |
|  | **Intervention: Education** | | |
| Mcgregor,  Mean | 4,767.62 | 5,752.35 | -984.73 |
| Boden,  Mean (SD / 95% CI) | 22,002 (17,919) | 23,331 (20,271) | -1,329 (95% CI: -2,221, -19,186) |
| Rolvings,  Mean (95% CI) | 53,629 (46,345, 60,858) | 53,719 (42,447, 63,727) | -91 (95% CI:-12,160, 12,342) |
| Furze,  Mean (SD/ 95% CI) | 10,024 | 10,422 | -398 |
|  | **Intervention: Nutrition + Physiotherapy + Education** | | |
| Ploussard,  Mean | 3,113 | 3,519 | -406 |
|  | **Intervention: Optimisation** | | |
| Partridge,  Mean (95% CI) | 5,067 | 6,335 | -1,218 (95% Cl: -6,665, 1,032) |
| Leeds (model) | 10,215 | 12,888 | -2,673 |

Cost are expressed in Pounds (GBP, £)

Adjusted for inflation to December 2022

SS = Pre- and post-operative nutrition, SC = Preoperative nutrition only, SC = Postoperative nutrition only, CC = No nutritional supplementation


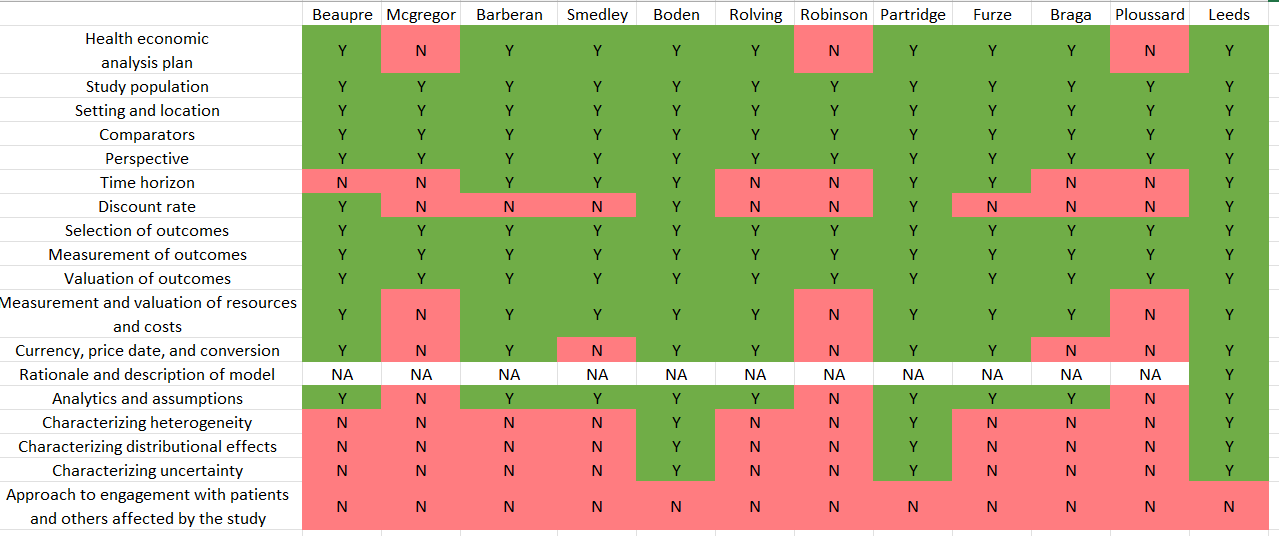


Figure A: CHEERS 2022 checklist.


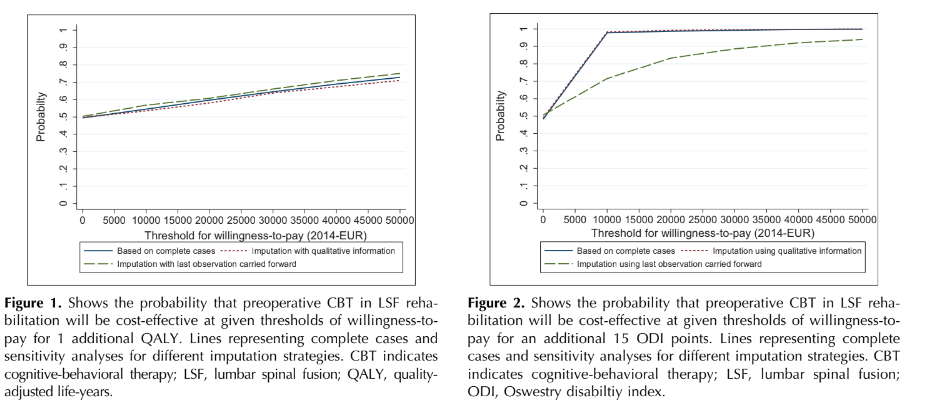


Figure B. Probability of preoperative cognitive behavior therapy (CBT) is cost-effective per QALY gained (left) and additional 15 ODI points (right). This graph is extracted from Rolving et al[[1]](https://paperpile.com/c/NrXafl/mnAQ).

1. [Rolving N, Nielsen CV, Christensen FB, Holm R, Bünger CE, Oestergaard LG. Preoperative cognitive-behavioural intervention improves in-hospital mobilisation and analgesic use for lumbar spinal fusion patients. BMC Musculoskelet Disord. 2016;17: 217. doi:](http://paperpile.com/b/NrXafl/mnAQ)[10.1186/s12891-016-1078-8](http://dx.doi.org/10.1186/s12891-016-1078-8)
